# Radiopalmar ganglion cysts: prevalence, morphology, and clinical significance in wrist MRI

**DOI:** 10.1007/s00330-024-10884-4

**Published:** 2024-07-03

**Authors:** Sophia S. Goller, Georg W. Kajdi, Georg C. Feuerriegel, Reto Sutter

**Affiliations:** Department of Radiology, Balgrist University Hospital, Faculty of Medicine, University of Zurich, Zurich, Switzerland

**Keywords:** Wrist, Radiopalmar ganglion cyst, Magnetic resonance imaging

## Abstract

**Objectives:**

To assess radiopalmar ganglion cysts’ (RPG) prevalence, morphology, and clinical significance in consecutive patients.

**Materials and methods:**

In this retrospective single-center study, two radiologists assessed the presence of RPG and morphologic features on wrist MRI. Radiopalmar complaints and scapholunate ligament (SLL) tears were evaluated.

**Results:**

A total of 1053 wrists in 909 patients (mean age 43.4 ± 15.5 years, 602 females) were evaluated. All 308 RPG (Group 1; 308 patients, 29.2%) originated from the palmar capsule; 49 were unilocular, 95 oligolocular, and 164 multilocular; 745 wrists had no RPG (Group 2; 601 patients). One hundred and twenty-six RPG showed internal debris. The mean diameter was 8.5 ± 5.6 mm (cranio-caudal) (1.0–32.9 mm), 8.0 ± 4.1 mm (medio-lateral) (1.0–31.9 mm), and 3.7 ± 2.3 mm (dorso-palmar) (0.4–16.0 mm). 168 RPG showed direct contact with the radial vascular bundle, 24 with the flexor carpi radialis tendon, and 123 with the flexor pollicis longus tendon. In Group 1, significantly more patients showed partial (82/308) [group 2: 45/745, *p* < 0.001] or complete SLL tears (22/308) [group 2: 20/745, *p* < 0.001]. Of the patients with RPG, 15.3% presented with radiopalmar complaints. Only the dorso-palmar RPG diameter was positively correlated with radiopalmar complaints (for readers 1 and 2: r_s_ = 0.66/0.61, *p* < 0.001, respectively), and the best dorso-palmar diameter cut-off value for the probability of having radiopalmar complaints was defined at 3 mm (area under the curve (AUC) 0.74). Other morphologic features were not eligible to discriminate symptomatic patients (AUC range 0.53–0.61).

**Conclusion:**

This study found RPG in 29% of patients, most of them asymptomatic. However, a dorso-palmar cyst diameter > 3 mm may be clinically significant.

**Clinical relevance statement:**

Radiopalmar ganglion cysts, observed in 29% of wrist MR examinations, are mostly asymptomatic, but those with a larger dorso-palmar diameter may be associated with radiopalmar complaints.

**Key Points:**

- *Radiopalmar ganglion cysts are found in 29% of patients undergoing wrist MRI.*
- *Most patients with evidence of radiopalmar ganglion cysts do not show radiopalmar symptoms (85%).*
- *A dorso-palmar cyst diameter > 3* *mm may be associated with radiopalmar complaints.*

## Introduction

Ganglion cysts are common benign soft tissue tumors with a synovial lining located anywhere in the appendicular skeleton but occur most frequently in the hand and wrist [1, 2]. The etiology and formation of ganglion cysts have not been fully elucidated; however, several theories exist, including mucinous degeneration, trauma, synovial herniation, and a one-way valve mechanism [3–5].

Ganglion cysts overlying a joint, ligament, or tendon sheath are reported to be most commonly found at the dorsal aspect of the wrist, especially in cases with scapholunate ligament (SLL) tears, where occult ganglion cysts are a possible cause of dorsal-sided wrist pain [6]. However, much less is known about ganglion cysts at the radiopalmar aspect of the wrist [7, 8]. A previous MRI study on 103 asymptomatic volunteers identified ganglion cysts in a total of 53 wrists and found the majority of them to be located at the palmar surface, reporting that most of them originated from the palmar capsule in the interval between the radioscaphocapitate (RSC) ligament and the long radiolunate ligament (LRL) [7], which we refer to in our study as “radiopalmar” ganglion cysts (RPG). The literature suggests that RPG frequently occur in asymptomatic volunteers and patients with wrist pain [1, 2, 6–14]. Still, their prevalence, morphologic features, and whether and when they are symptomatic remain unclear.

With this background, this study evaluated the prevalence, morphology, and clinical implications of RPG in a large cohort of symptomatic patients with MRI.

## Materials and methods

### Patient selection

This retrospective single-center study was approved by the local institutional review board (Cantonal Ethics Committee Zurich) and conducted according to the principles of the Declaration of Helsinki and national ethical standards. All patients included in the data collection have given written informed consent that allows their health-related data to be used for research purposes.

**Fig. 1:**
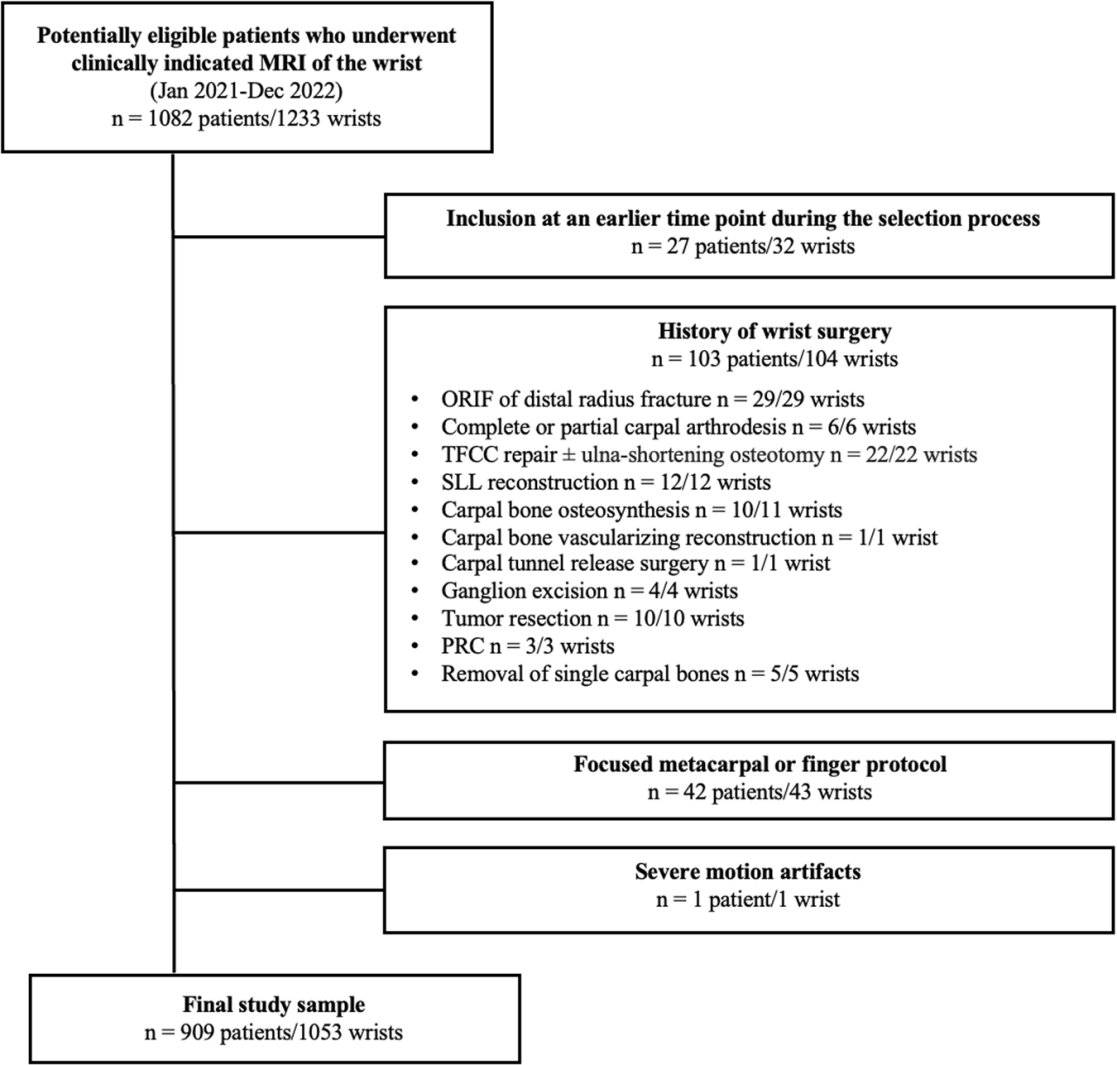
Flowchart illustrating the patient selection process. From *n* = 1082 potentially eligible patients, 173 patients had to be excluded during the selection process, resulting in a final study sample of 1053 wrists in 909 patients. ORIF, open reduction and internal fixation; PRC, proximal row carpectomy; SLL, scapholunate ligament; TFCC, triangular fibrocartilage complex

The institutional picture archiving and communication system (PACS) was reviewed for patients who underwent wrist MRI for various clinical indications, such as radial-sided, ulnar-sided or central, diffuse, or vaguely localized wrist pain with and without trauma, arthritis, (teno-)synovitis, or crystalline arthropathy, osteoarthritis, avascular necrosis/ pseudarthrosis, carpal tunnel or other nerve bottle neck syndromes, tumors and tumor-like lesions, and clinically suspected and/or ultrasound-proven RPG (detailed information are given in Supplementary Table 1) between January 2021 and December 2022 (*n* = 1233 wrists in 1082 patients, Fig. 1). Exclusion criteria were prior wrist surgery, incomplete depiction of the wrist (due to protocols focused on fingers or metacarpals), and MRI studies with severe motion artifacts.

### Magnetic resonance imaging

MRI of the wrist was conducted on either a 1.5-T unit (Magnetom Sola, Siemens Healthineers) (*n* = 572 wrists, 54.3%) or a 3-T unit (Magnetom Vida, Siemens Healthineers) (*n* = 481 wrists, 45.7%) using a dedicated 16-channel phased-array wrist coil. All patients underwent routine wrist imaging protocols in supine and head-first positions. Depending on the individual clinical indication, the examinations were performed as noncontrast MRI (*n* = 226 wrists, 21.5%), as MRI with intravenous gadolinium contrast (*n* = 517 wrists, 49.1%), or as direct MR arthrography (*n* = 310 wrists, 29.4%). Accordingly, the sequences varied depending on the scanner (1.5 T vs. 3 T) and the selected type of MRI examination (Supplementary Table 2). For scans with intravenous contrast, 0.1 mL gadolinium per kg of body weight (Gadovist 1.0 mmol/mL, Bayer) was applied. For direct MR arthrography, a 2 mL mixture of 2/3 gadoteric acid (Artirem 0.0025 mmol/mL, Guerbet) and 1/3 iodinated contrast medium (Iopamiro 300 mg/mL, Bracco) was injected into the midcarpal as well as distal radioulnar compartment under fluoroscopic guidance using strict aseptic precautions and 24-gauge needles.

### Image analysis

Image analysis was done using a commercially available picture archiving and communication system (PACS) workstation (Merlin, Phoenix-PACS). All MRI studies were anonymized and independently reviewed by two musculoskeletal fellowship-trained radiologists (S.S.G. and G.W.K., with five and six years of experience, respectively). Examinations were analyzed in random order, whereby both readers were blinded to clinical data.

**Fig. 2:**
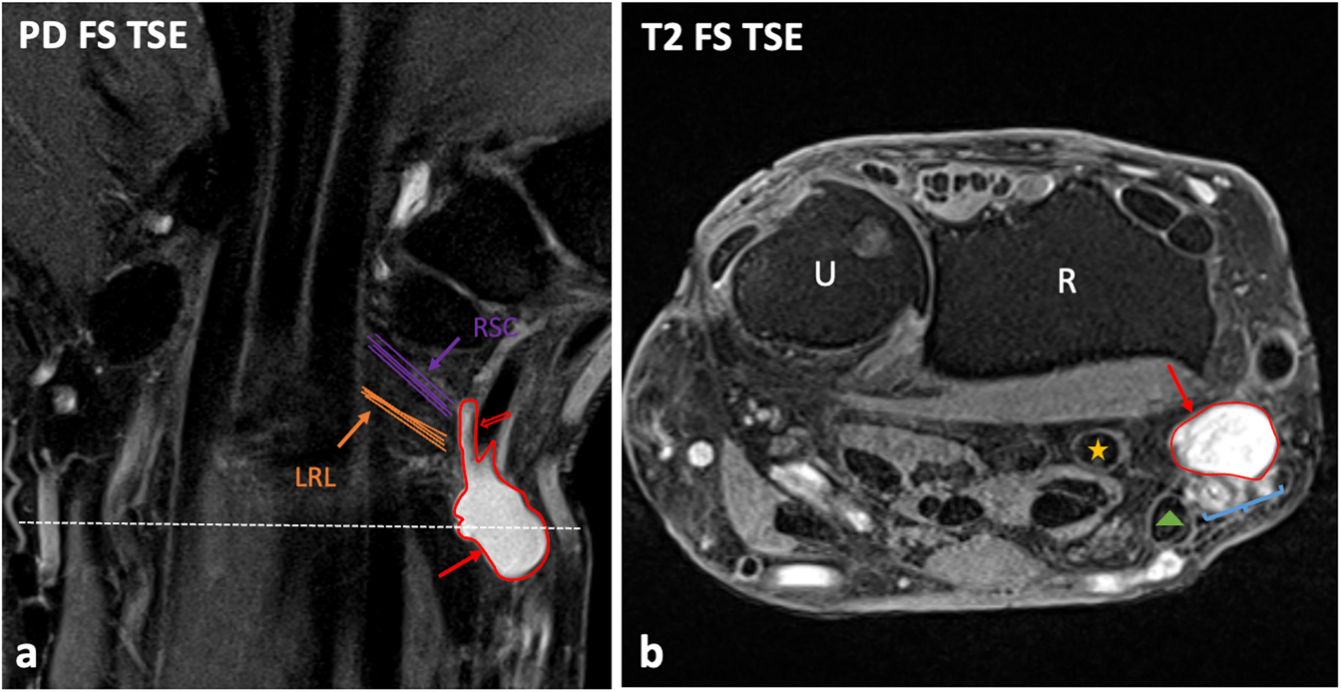
Patient example illustrating the MR anatomy of a radiopalmar ganglion cyst (RPG). Coronal fat-saturated (FS) proton-density (PD)-weighted image (**a**) and transversal FS T2-weighted turbo-spin-echo (TSE) image at the level of the distal radioulnar joint (dashed line in image A) (**b**) of a left wrist show an RPG (red outlines and arrows) with typical characteristics. The RPG originates with a thin neck (outline arrow, **a**) from the palmar capsule in the interval between the radioscaphocapitate (RSC) ligament and the long radiolunate ligament (LRL), whose fiber orientations are illustrated in image **a** (lines and arrows). RPG might have direct contact with adjacent structures, including the radial vascular bundle (curly brackets, **a**), the flexor carpi radialis (FCR) tendon (triangle, **a**), and the flexor pollicis longus (FPL) tendon (asterisk, **a**). R, radius; U, ulna

**Fig. 3:**
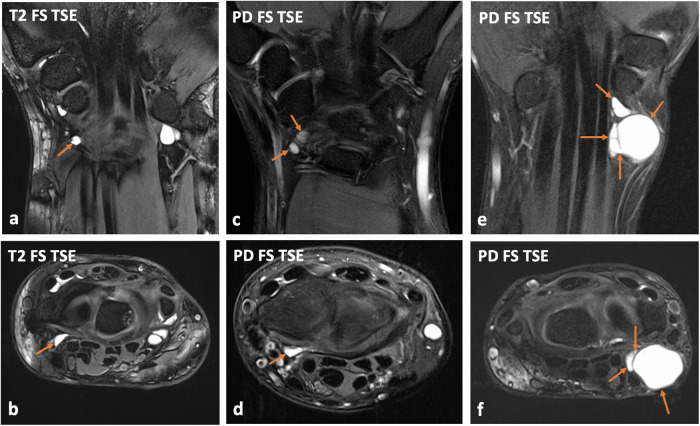
Patient examples illustrating the assessment of radiopalmar ganglion cyst (RPG) loculation. Coronal (**a**) and transversal (**b**) T2-weighted turbo-spin-echo (TSE) images of a right wrist show a “unilocular” radiopalmar ganglion cyst (RPG), which consists of a single cavity (arrows). Coronal (**c**) and transversal (**d**) fat-saturated (FS) proton density (PD)-weighted TSE images of a right wrist depict an “oligolocular” RPG, which consists of two separated cavities (arrows, **c**), which were connected via a narrow cyst neck (not shown). It should be noted that the more cranially located cyst contained debris (shown on the axial image, **d**), while the more inferiorly located one showed a fluid-isointense signal. Coronal (**e**) and transversal (**f**) FS PD-weighted images of a left wrist illustrate a “multilocular” RPG, which consists of multiple separated cavities (arrows)

**Fig. 4:**
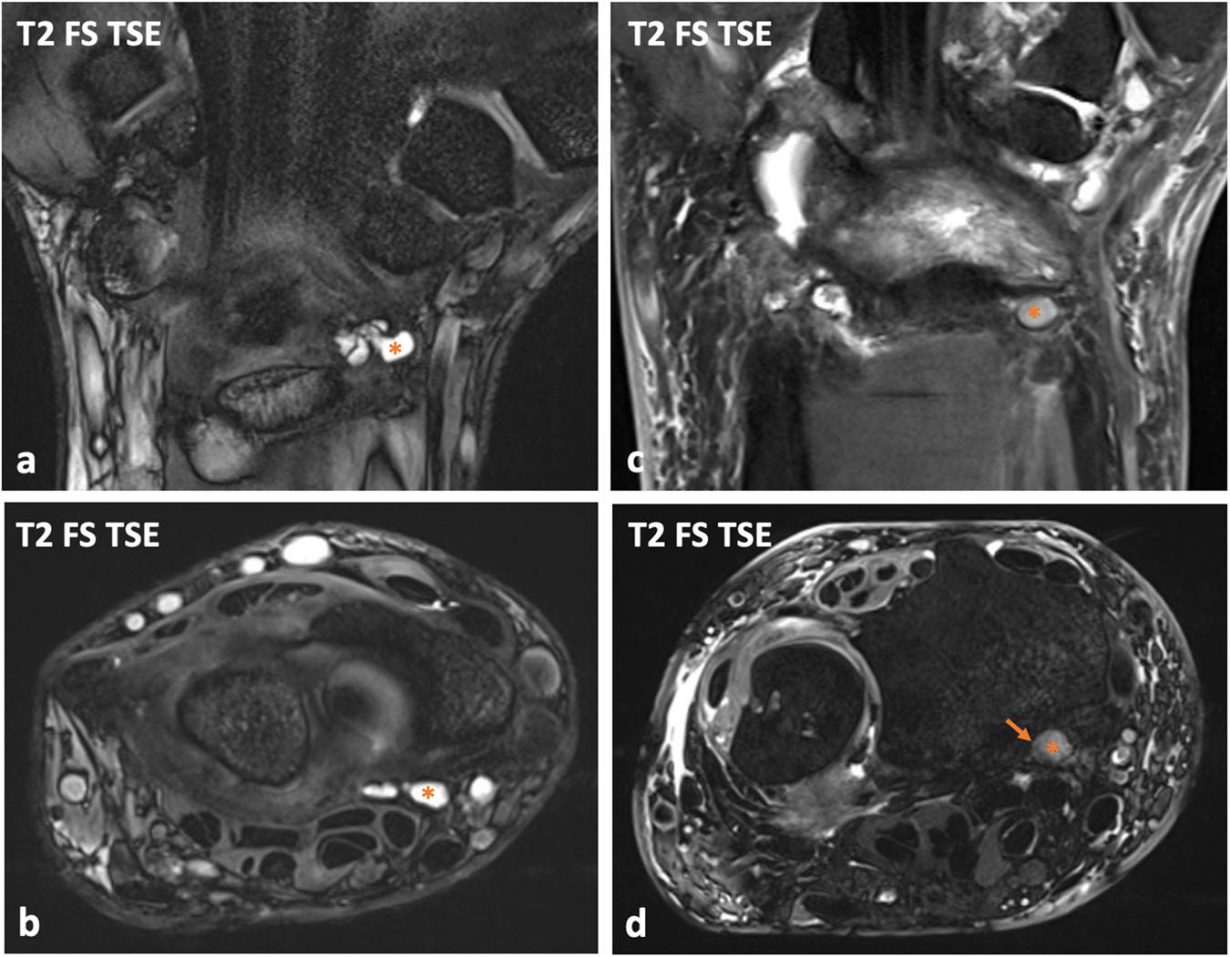
Patient examples illustrating debris assessment within radiopalmar ganglion cysts (RPG). Coronal (**a**) and transversal (**b**) fat-saturated (FS) T2-weighted turbo-spin-echo (TSE) images of a left wrist delineate a multilocular RPG, which has a fluid-isointense signal and does not contain debris (asterisks). In contrast, a small unilocular RPG of a right wrist is relatively hypointense on the coronal (**c**) and transversal (**d**) FS T2-weighted TSE images (asterisks) and suggests layering within the ganglion cyst (arrow, **d**), wherefore it was rated as containing debris

**Fig. 5:**
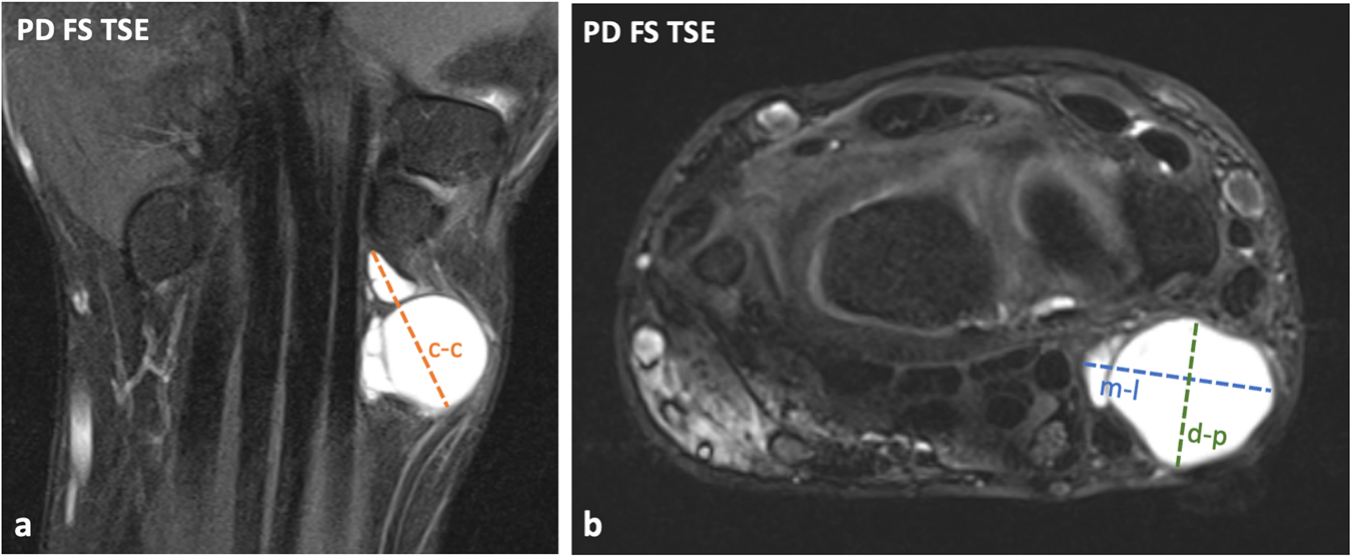
Patient example illustrating the radiopalmar ganglion cyst (RPG) diameter measurement. Coronal (**a**) and transversal (**b**) fat-saturated (FS) proton density (PD)-weighted TSE images of a left wrist (same patient as in Fig. 2) show a multilocular RPG. For each MRI, the largest adjustable ganglion cyst diameters in the cranio-caudal (c-c), medio-lateral (m-l), and dorso-palmar (d-p) directions were determined by sampling through all spatial planes

**Fig. 6:**
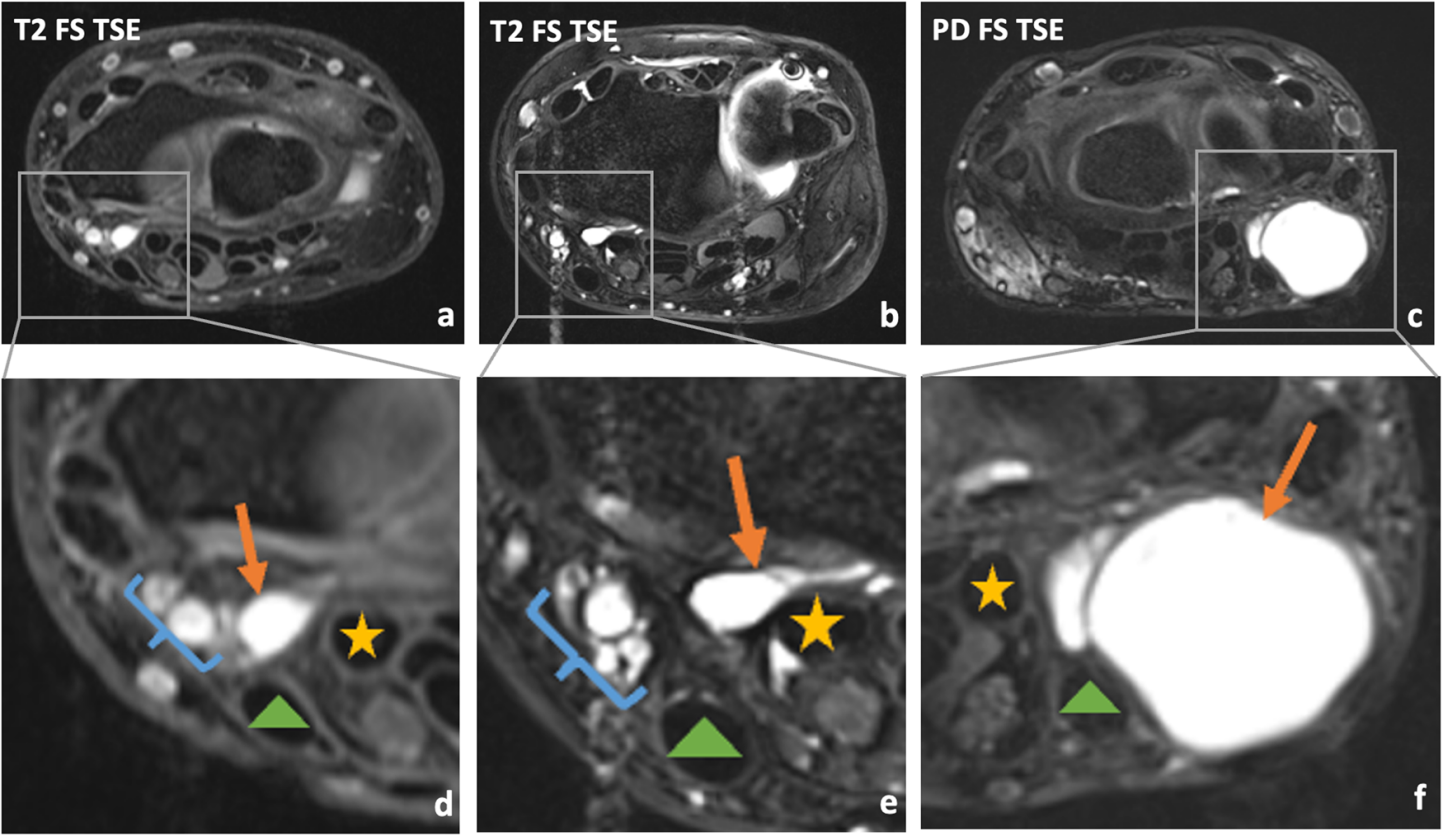
Patient examples illustrating the determination of direct contact of radiopalmar ganglion cysts (RPG) and adjacent structures: Radial vascular bundle, flexor carpi radialis (FCR) tendon, and flexor pollicis longus (FPL) tendon. Transversal fat-saturated (FS) T2-weighted turbo-spin-echo (TSE) image (**a**) and magnified image of the radiopalmar area (**d**) show a right wrist with an unilocular RPG (arrow), which is in direct contact with the radial vascular bundle (curly brackets), but not with the FCR (triangle) or the FPL (asterisk) tendon. Transversal FS T2-weighted TSE image (**b**) and magnified image of the radiopalmar area (**e**) of a right wrist depict an oligolocular RPG (arrow), which has direct contact with the FPL tendon (asterisk). A multilocular RPG (arrow) is illustrated in the transversal FS PD-weighted image (**c**) and magnified image of the radiopalmar area (**f**) of a left wrist (same patient as in Figs. 2 and 4), which has direct contact with the FCR tendon (triangle) and invades the radial vascular bundle

For each scan, readers had to indicate whether or not an RPG was present (the MR anatomy of an RPG is described in Fig. 2). Following previous literature [7], the criteria for the diagnosis of a ganglion cyst were 1) a well-defined mass showing 2) a low signal on T1-weighted images, 3) a high, more or less fluid-equivalent signal on T2- or proton-density (PD)-weighted images, 4) contact with a joint capsule or tendon sheath, and, in distinction from blood vessels, 5) neither a tubular shape nor pulsation artifacts. If an RPG was detected, each reader had to determine its loculation, which could be either unilocular (consisting of a single cavity), oligolocular (2–3 cavities), or multilocular (> 3 cavities) (Fig. 3), evaluate whether or not there was debris within the ganglion (debris was diagnosed when there was hypointensity relative to fluid-signal on T2- or PD-weighted sequences and/or layering and/or floating particles within the ganglion) (Fig. 4), measure its maximum diameter in all three spatial planes (craniocaudal, medio-lateral, and dorso-palmar) (Fig. 5), and indicate whether it was in direct contact with the radial vascular bundle, the flexor carpi radialis (FCR) tendon, or the flexor pollicis longus (FPL) tendon using fluid-sensitive sequences (Fig. 6). To make the diameter measurement as reliable as possible, exclusively PD-weighted sequences were used for this purpose, as they were acquired in three spatial planes. In addition, one of the readers (S.S.G.) evaluated all MR scans for the presence of a potential SLL tear and systematically assessed all MR scans with regard to potential concomitant pathologies other than RPG that could likely cause radiopalmar wrist complaints, including 1) bone marrow edema (distal radius, scaphoid, trapezium, trapezoid, capitate), 2) signs of arthritis, (teno-)synovitis, or crystalline arthropathy, 3) osteoarthritis (distal radioulnar joint, radiocarpal joint), 4) avascular necrosis or pseudarthrosis, 5) carpal tunnel syndrome, and 6) tumors and tumor-like lesions (both benign and malignant). Further, it was recorded according to the information in the clinical information system by the referring physician at the time of the indication (max. four weeks before the MRI) whether the patients had radiopalmar complaints such as pain, swelling, motion restriction, or a combination of these symptoms.

### Statistical analysis

All statistical analyses were performed in SPSS Statistics (v. 29, IBM Corporation).

Non-normal distribution of data was assessed graphically and analytically using Quantile-Quantile plots and the Shapiro-Wilk test. Wrists were divided into Group 1 (RPG) and Group 2 (no RPG). In addition to descriptive statistics, the Mann-Whitney-U-test and the Chi-square-test were performed to assess differences between the groups. Receiver operating characteristic (ROC) curves and the Youden index were used to evaluate binary classifier models’ performance and determine cut-off values. Correlation coefficients (r_s_) were calculated by non-parametric correlation analysis (Spearman’s correlation). The inter-reader agreement was analyzed by kappa statistics (Cohens’ κ) and intraclass correlation coefficients (ICC). The level of agreement was categorized as follows [9]: 0.0 = poor, 0.01–0.20 slight, 0.21–0.40 = fair, 0.41–0.60 = moderate, 0.61–0.80 = substantial, 0.81–1.00 = almost perfect agreement. All statistical tests were performed two-sided, and a level of significance (α) of 0.05 was used.

## Results

### Demographics

A total of 1053 wrists in 909 patients (mean age 43.4 ± 15.5 years, 602 females, Table 1) were included in the study. A total of 308 wrists (29.2%) showed an RPG on MRI (Group 1308 patients), while 745 wrists (70.8%) did not (Group 2, 601 patients). Although there was a statistical significance in age in the two groups, this was not clinically significant (Group 1: 41.8 ± 14.9 vs. Group 2: 44.0 ± 15.6 years, *p* = 0.027). There were no significant differences regarding gender distribution (165/308 vs. 437/745 females, *p* = 0.13) or affected side (189/308 vs. 418/745 right wrists, *p* = 0.12).

**Table 1:** Comparison of patient characteristics between the groups

	RPG	No RPG	*p*-value ^a^
Wrists/patients (*n*)	308/308	745/601	
Female (*n*, %)	165 (53.6)	437 (58.7)	0.13
Age (years) ^b^	41.8 ± 14.9	44.0 ± 15.6	**0.027**
Right wrist (*n*, %)	189 (61.4)	418 (56.1)	0.12
Radiopalmar complaints * (*n,* %)	47 (15.3)	16 (2.7)	**<** **0.001**
Scapholunate ligament (*n*, %)			
Intact	204 (66.2)	680 (91.3)	**<** **0.001**
Partial tear	82 (26.6)	45 (6.0)	**<** **0.001**
Partial tear, dorsal part	29 (9.4)	10 (1.3)	
Partial tear, membranous part	29 (9.4)	21 (2.8)	
Partial tear, palmar part	10 (3.2)	6 (0.8)	
Partial tear, ≥ 2 parts involved	14 (4.5)	8 (1.1)	
Complete tear	22 (7.1)	20 (2.7)	**<** **0.001**
RPG characteristics			
Loculation (*n*, %)			
Unilocular (one single cavity)	49 (15.9)	N/A	
Oligolocular (> 1 ≤ 3 cavities)	95 (30.8)	N/A	
Multilocular (> 3 cavities)	164 (53.2)	N/A	
Debris (*n*, %)	126 (40.9)	N/A	
Diameter [mm^2^]			
Cranio-caudal direction	8.5 ± 5.6	N/A	
Medio-lateral direction	8.0 ± 4.1	N/A	
Dorso-palmar direction	3.7 ± 2.3	N/A	
Contact with adjacent structures (*n*, %)			
Contact with radial vascular bundle	168 (54.5)	N/A	
Contact with flexor carpi radialis tendon	24 (7.8)	N/A	
Contact with flexor pollicis longus tendon	123 (39.9)	N/A	

^a^ Significant results (*p* < 0.05) are bolded, ^b^ Data is given as mean ± standard deviation, * Including pain, swelling, motion restriction, or a combination of these symptoms.

*N/A* not applicable, *RPG* radiopalmar ganglion cysts

### Morphology

All of the 308 RPG originated from the palmar capsule in the interval between the RSC and LRL ligament; 49 (15.9%) were unilocular, 95 (30.8%) oligolocular, and 164 (53.2%) multilocular. A total of 126 (40.9%) RPG showed internal debris. The mean RPG diameter was 8.5 ± 5.6 mm in the cranio-caudal direction (minimum-maximum diameter: 1.0–32.9 mm), 8.0 ± 4.1 in the medio-lateral direction (minimum-maximum diameter: 1.0–31.9 mm), and 3.7 ± 2.3 mm in the dorso-palmar direction (minimum-maximum diameter: 0.4–16.0 mm). A total of 168/308 RPG (54.5%) showed direct contact with the radial vascular bundle, whereas 24 RPG (7.8%) showed direct contact with the FCR tendon, and 123 RPG (39.9%) with the FPL tendon, respectively.

### Clinical associations

Significantly more patients with RPG (47/308, 15.3%) showed radiopalmar complaints, including pain, swelling, motion restriction, or a combination of these symptoms when compared with patients without RPG (16/601, 2.7%, *p* < 0.001). In Group 1, significantly more patients had either a partial (82/308, 26.6%) (Group 2: 45/745, 6.0%, *p* < 0.001) or a complete tear (22/308, 7.1%) (Group 2: 20/745, 2.7%, *p* < 0.001) of the SLL, compared to those without RPG in Group 2. However, an SLL tear was unsuitable for differentiating between symptomatic and asymptomatic patients (AUC 0.62). Besides SLL tears, the evaluation of concomitant findings at the radiopalmar wrist revealed no significant differences between Group 1 and Group 2: A total of 19/308 patients (6.2%) in Group 1 presented with bone marrow edema at the radiopalmar site of the wrist, while bone marrow edema was present in 55/601 patients (9.2%) in Group 2 (*p* = 0.10). Arthritis, (teno-)synovitis, or crystalline arthropathy, was present in 31/308 patients (10.1%) in Group 1 and in 85/601 patients (14.1%) in Group 2 (*p* = 0.07). In Group 1, 4/308 patients (1.3%) had osteoarthritis of the distal radioulnar joint or the radiocarpal joint, while in Group 2, osteoarthritis was present in 9/601 patients (1.5%) (*p* = 0.81). A total of 2/308 patients (0.6%) in Group 1 had avascular necrosis or pseudarthrosis, whereas in Group 2, 10/601 patients (1.7%) were affected by this condition (*p* = 0.21). Carpal tunnel syndrome was found in 4/308 patients (1.3%) in Group 1 and 11/601 patients (1.8%) in Group 2 (*p* = 0.55). Tumors and tumor-like lesions (both benign and malignant) were seen in 5/308 patients (1.6%) in Group 1 and 12/601 patients (2.0%) in Group 2 (*p* = 0.70).

The dorso-palmar RPG diameter was positively correlated with the presence of radiopalmar complaints (r_s_ = 0.66/0.61 for readers 1 and 2, respectively, *p* < 0.001), whereby ROC curves showed a reliable dorso-palmar diameter cut-off value for the probability of having radiopalmar complaints at 3 mm (AUC 0.74; 95%CI: 0.66, 0.82) (Supplementary Fig. 1). For the cranio-caudal and medio-lateral RPG diameters, there was no significant correlation with radiopalmar complaints (cranio-caudal diameter: r_s_ = 0.09/0.74 for readers 1 and 2, *p* = 0.06/0.97 and medio-lateral diameter: r_s_ = 0.051/0.045 for readers 1 and 2, *p* = 0.19/0.43, respectively).

Furthermore, neither direct contact of an RPG with the radial vascular bundle, the FCR tendon, nor the FPL tendon was suitable for discriminating between patients with and without radiopalmar complaints (AUC 0.54, 0.61, and 0.53, respectively). Likewise, neither the evidence of debris nor the loculation (AUC 0.57 and 0.55) were appropriate for discriminating radiopalmar-sited symptomatic and asymptomatic patients.

### Inter-reader agreement

The inter-reader agreement between the two readers was almost perfect for the assessment of internal debris (Cohen’s κ 0.90; 95%CI: 0.85, 0.95) and RPG loculation (κ 0.95; 95%CI: 0.91, 0.98), as well as for direct contact with the radial vascular bundle (κ 0.93; 95%CI: 0.90, 0.97), the FCR tendon (κ 0.91; 95%CI: 0.81, 0.99), and the FPL tendon (κ 0.95; 95%CI: 0.92, 0.99). A substantial agreement was found for the assessment of the cranio-caudal RPG diameter (ICC 0.75; 95%CI: 0.75, 0.75), as well as the dorso-palmar diameter (ICC 0.77; 95%CI: 0.76, 0.78), while an almost perfect agreement was found for the measurement of the medio-lateral RPG diameter (ICC 0.82; 95%CI: 0.82, 0.82).

## Discussion

In this study, we demonstrated that radiopalmar ganglion cysts (RPG) can be found in 29% of patients undergoing wrist MRI and are asymptomatic with regard to radiopalmar complaints in most cases (85%). However, the dorso-palmar RPG diameter was associated with radiopalmar complaints, mainly when larger than the cut-off value of 3 mm.

With 29%, the prevalence of RPG in our cohort was slightly higher than that of wrist ganglia in general, as reported in a previous MRI study on 625 wrists by El-Noueam, where the authors found ganglion cysts in 19%. However, it is unclear from their data how many of the detected ganglion cysts were in a radiopalmar localization [10]. In contrast, in pediatric cohorts, the prevalence of wrist ganglion cysts was reported to be higher, with 36 and 56%, respectively [11, 12]. Accordingly, ganglia might be more common in children and adolescents than adults. In our study, the RPG group was significantly younger than the cohort without evidence of an RPG, though this difference was numerically minimal (41.8 vs. 44.0 years); however, only 53/909 patients in our study were 18 years or younger; therefore, our results cannot support this hypothesis. Besides, some previous studies analyzed the prevalence of ganglion cysts in both the palmar and dorsal side of the wrist in asymptomatic volunteers and reported a prevalence of up to 50% [7, 13].

More than half of the RPG in our cohort were multilocular (53%), whereas 41% showed internal debris. In contrast, most ganglion cysts analyzed in an asymptomatic population were described as simple, with relatively few septations/lobules and minimal debris [7]. This difference might be explained by the fact that we assessed symptomatic patients instead of asymptomatic individuals. However, neither the degree of loculation nor the evidence of debris was eligible to discriminate between patients with and without radiopalmar complaints.

In terms of diameter, the ganglion cysts in our study did not significantly differ from wrist ganglia reported in previous studies. While the RPG in our cohort showed mean diameters of approximately 9 mm in cranio-caudal, 8 mm in medio-lateral, and 4 mm in dorso-palmar direction, a mean long axis of 8 mm and a mean short axis of 3 mm was reported for ganglion cysts analyzed in a previous MRI study [7]. Similarly, a mean diameter of 7 mm was found by Bracken et al, who investigated pediatric wrist MR scans [11].

Although most RPG were asymptomatic with regard to radiopalmar complaints (85%), we found radiopalmar-sited conditions, including pain, swelling, motion restriction, and combinations of these symptoms, significantly more often in patients with RPG than in those without RPG (15% vs. 3%). Looking for reasons, our study demonstrated that neither direct contact of an RPG with the radial vascular bundle, the FCR tendon, or the FPL tendon nor the presence of SLL tears is suitable for discriminating between patients with and without radiopalmar complaints. Interestingly, however, the dorso-palmar RPG diameter was positively correlated with radiopalmar complaints, and we could define a reliable cut-off value at 3 mm. Considering that modern high-resolution MRI allows an accurate visualization of even small ganglion cysts and definitive treatment options like cyst aspiration and arthroscopic removal may be associated with surgical complications and recurrence [5], this finding seems essential to avoid unnecessary interventions whose indication is based solely on accidentally detected ganglion cysts. On the other hand, it would be equally unfavorable not to treat RPG, which may be the underlying cause of pain.

Another interesting aspect of our study is that the prevalence of SLL tears was significantly higher in patients with RPG evidence than in those without. This observation was made for partial and complete tears; however, SLL tears were not eligible as a discriminator for symptomatic patients. Instead, this finding might be explained by one of the manifold theories on the origin and pathophysiology of ganglion cysts that postulate that ganglion cysts are secondary manifestations of underlying periscaphoid ligament injuries [14]. Strengthening this hypothesis, a previous study found the majority of arthroscopically-treated dorsal wrist ganglia in patients with concomitant SLL and lunotriquetral ligament tears, wherefore the authors concluded that increased intercarpal laxity might contribute to ganglion cyst formation [15]. However, today, the correlation between SLL tears and wrist ganglia is controversial, as many competing theories exist [3–5]. For example, Rizzo et al found no cases of SLL instability among patients treated for dorsal-sided wrist ganglia [16] and, similarly, in the study by Lowden and colleagues, most wrist ganglia detected on MRI were not accompanied by ligamentous pathologies [7].

Several limitations of this study need to be addressed. First, the study design was retrospective; patients were not randomized, nor was there a control group. Besides, only symptomatic patients examined at a single institution were included. Further, as this was a retrospective study, patients’ clinical information had been documented by the referring physician at the time of the indication before the MRI and, therefore, could not be verified. Second, besides RPG, there might be some bias due to concomitant pathologies that could likely cause radiopalmar complaints. However, we did not see significant differences in various analyzed co-pathologies between patients with RPG and those without, which is why it can be assumed that this possible bias should not influence the results too strongly. Logistic regression models might, among other things, have been advantageous in minimizing this potential bias. However, the required G*Power was not achieved. Third, the assessment of RPG was done solely on MRI, and no surgical confirmation was available. However, due to its excellent soft tissue contrast, MRI represents the imaging modality of choice for the visualization of even small ganglion cysts.

Further, MRI was performed using different scanners, field strengths, and protocols, which might limit the generalizability and comparability of our results, for example, due to varying sensitivities for specific pathologies, e.g., SLL tears. Fourth, we did not analyze ganglion cysts at any localization other than radiopalmar. Fifth, due to the study’s retrospective nature and lack of data, we could not assess patients’ treatments and outcomes sufficiently. However, so far, our study comprises the largest cohort that has analyzed RPG in consecutive patients.

In conclusion, this study indicates a prevalence of radiopalmar ganglion cysts (RPG) of 29% in consecutive patients, while most were asymptomatic. However, a dorso-palmar cyst diameter > 3 mm may be clinically significant.

## Supplementary information

Electronic Supplementary Material

## Abbreviations

AUC: Area under the curve
FCR: Flexor carpi radialis
FPL: Flexor pollicis longus
LRL: Long radiolunate ligament
ROC: Receiver operating characteristic
RPG: Radiopalmar ganglion cyst(s)
RSC: Radioscaphocapitate ligament
SLL: Scapholunate ligament
