# Supplementary material for: Radiopalmar ganglion cysts: prevalence, morphology, and clinical significance in wrist MRI
Source: Eur Radiol. 2024 Jul 3;34(12):7869–77. doi: 10.1007/s00330-024-10884-4 (PMC11557648; doi:10.1007/s00330-024-10884-4)
Supplement: Supplementary file 1 — Electronic Supplementary Material [file 330_2024_10884_MOESM1_ESM.pdf]

Radiopalmar ganglion cysts: Prevalence, morphology, and clinical significance in wrist MRI
ELECTRONIC SUPPLEMENTARY MATERIAL

Supplementary Table 1: MRI indications.

	RPG	No RPG
**MRI indication (n, %)**		
Radial-sided wrist pain without trauma	44 (14.3)	54 (7.2)
Ulnar-sided wrist pain without trauma	47 (15.3)	68 (9.1)
Central, diffuse, or vaguely localized wrist pain without trauma	23 (7.5)	63 (8.5)
Radial-sided wrist pain with trauma	29 (9.4)	65 (8.7)
Ulnar-sided wrist pain with trauma	36 (11.7)	67 (9.0)
Central, diffuse, or vaguely localized wrist pain with trauma	30 (9.7)	43 (5.8)
Arthritis, (teno-)synovitis, or crystalline arthropathy	49 (15.9)	269 (36.1)
Osteoarthritis	8 (2.6)	11 (1.5)
Avascular necrosis/pseudarthrosis	3 (1.0)	17 (2.3)
Carpal tunnel syndrome/other nerve bottleneck syndromes	8 (2.6)	18 (2.4)
Tumors and tumor-like lesions (both benign and malignant) including vascular malformations and ganglion cysts in other locations than radiopalmar	6 (1.9)	43 (5.8)
Clinically suspected and/or ultrasound-proven RPG	25 (8.1)	0 (0)

RPG, radiopalmar ganglion cysts.

Supplementary Table 2: Sequence parameters at the 1.5T and 3T scanner.

**Non-contrast 1.5T MRI**					
Sequence	T1 TSE cor	PD TSE FS cor	PD TSE FS sag	PD TSE FS tra	T2 TSE tra
TR [ms]	654	3300	3500	3740	3600
TE [ms]	13	21	35	38	79
Bandwidth [Hz/px]	160	70	160	150	200
Slices [n]	19	19	20	22	22
Slice thickness [mm]	2	2	3	3	3
Spacing [mm]	2.4	2.4	3.6	3.3	3.3
Matrix	256x320	288x320	256x320	256x320	256x320
FOV [mm]	100	90	100	100	100
TA [min:s]	1:02	4:14	2:30	2:52	3:32

**1.5T MRI with intravenous contrast**						
Sequence	T1 TSE cor	PD TSE FS cor	PD TSE FS sag	T2 TSE tra	PD TSE FS tra	ce T1 TSE FS tra
TR [ms]	654	3300	3500	3600	3740	549
TE [ms]	13	21	35	79	38	13
Bandwidth [Hz/px]	160	70	160	200	150	190
Slices [n]	19	19	20	22	22	30
Slice thickness [mm]	2	2	3	3	3	2
Spacing [mm]	2.4	2.4	3.6	3.3	3.3	2.4
Matrix	256x320	288x320	256x320	256x320	256x320	209x256
FOV [mm]	100	90	100	100	100	90
TA [min: s]	1:02	4:14	2:30	3:32	2:52	3:02

**1.5T MR arthrography**					
Sequence	T1 TSE cor*	PD TSE FS cor*	PD TSE FS sag*	T2 TSE tra*	PD TSE FS tra*

TR [ms]	589	2950	3500	3600	3740
TE [ms]	13	21	35	79	38
Bandwidth [Hz/px]	160	70	160	200	150
Slices [n]	17	17	19	22	22
Slice thickness [mm]	2	2	3	3	3
Spacing [mm]	2.4	2.4	3.6	3.3	3.3
Matrix	256x320	288x320	256x320	256x320	256x320
FOV [mm]	100	90	100	100	100
TA [mins]	1:52	3:47	2:30	3:32	2:52

Non-contrast 3T MRI

Sequence	T1 TSE cor	PD TSE FS cor	PD TSE FS sag	PD TSE FS tra	T2 TSE tra
TR [ms]	500	3000	3500	3800	5960
TE [ms]	14	42	27	39	61
Bandwidth [Hz/px]	372	252	199	150	180
Slices [n]	18	18	24	26	26
Slice thickness [mm]	2	2	3	2.5	2.5
Spacing [mm]	2.2	2.2	3.3	2.75	2.75
Matrix	307x384	280x352	307x384	176x352	160x320
FOV [mm]	90	90	100	62.5	62.5
TA [mins]	1:13	3:15	1:45	2:20	0:59

3T MRI with intravenous contrast

Sequence	PD TSE FS cor	T1 TSE cor	PD TSE FS sag	T2 TSE tra	PD TSE FS tra	ce T1 TSE FS tra
TR [ms]	3000	500	3500	5960	3800	650
TE [ms]	42	14	27	61	39	13
Bandwidth [Hz/px]	245	370	255	180	150	365
Slices [n]	18	18	24	26	26	26
Slice thickness [mm]	2	2	3	2.5	2.5	2.5
Spacing [mm]	2.2	2.2	3.3	2.75	2.75	2.75

Matrix	280x352	307x384	307x384	160x320	176x352	320x336
FOV [mm]	90	100	100	62.5	62.5	105
TA [mins]	3:15	1:13	1:45	0:59	2:20	2:06

**3T MR arthrography**					
Sequence	PD TSE FS cor*	T1 TSE cor*	PD TSE FS sag*	PD TSE FS tra*	T2 TSE tra*
TR [ms]	3000	500	3500	3800	5960
TE [ms]	42	14	27	39	61
Bandwidth [Hz/px]	250	370	255	150	150
Slices [n]	18	18	24	26	26
Slice thickness [mm]	2	2	3	2.5	2.5
Spacing [mm]	2.2	2.2	3.3	2.75	2.75
Matrix	280x352	307x384	307x384	176x352	160x320
FOV [mm]	90	90	100	62.5	62.5
TA [min:s]	1:36	0:56	1:24	2:20	1:05

Ce, contrast-enhanced; cor, coronal; Hz, Hertz; FS, fat-saturated; FOV, field of view; PD, proton density; sag, sagittal; px, pixel; TA, acquisition time; TE, echo time; TR, repetition time; tra, transversal; TSE, turbo-spin-echo.
** Sequences acquired after the administration of intra-articular contrast medium.*

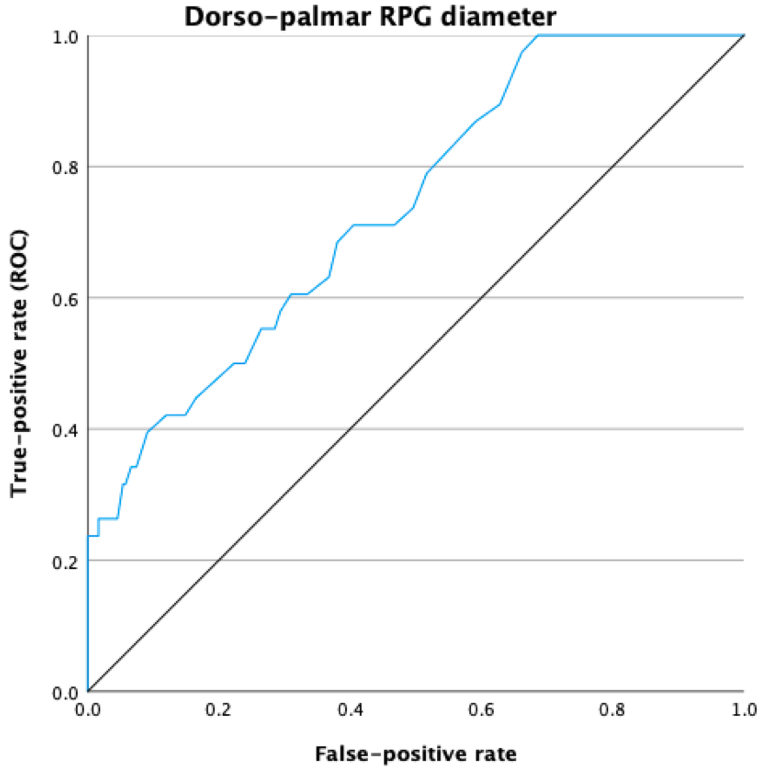

Supplementary Figure 1: Receiver operating characteristic (ROC) curve for using the radiopalmar ganglion cyst's (RPG) dorso-palmar diameter to distinguish patients with radiopalmar-sited complaints from those without.

The area under the curve (AUC) was 0.74 (95% CI: .66, .82). The best cut-off value was found at 3 mm with a sensitivity of .71 and specificity of .59.
